# Medication-associated tardive dyskinesia risks in real-world pharmacovigilance: a disproportionality analysis based on FAERS

**DOI:** 10.1192/j.eurpsy.2026.10634

**Published:** 2026-07-31

**Authors:** L. Zhang, H. Li

**Affiliations:** Shanghai Mental Health Center, Shanghai, China

## Abstract

**Introduction:**

Tardive dyskinesia, or TD, is a drug-induced movement disorder, linked with long-term use of dopamine receptor blockers. TD imposes a substantial healthcare burden, with higher hospitalization and emergency visits, higher medical costs, and increased psychiatric and physical comorbidities.

**Objectives:**

This study aims to identify drugs associated with TD risks beyond established agents such as metoclopramide using the FDA Adverse Event Reporting System (FAERS) database, which is a public, quarterly-updated, spontaneous reporting system for adverse drug events.

**Methods:**

TD reports were extracted from the FAERS database (Q1 2004 to Q4 2024). Four disproportionality methods were appllied to identify statistically significant TD risk signals. Sensitivity analyses utilizing adjusted RORs (aRORs) were conducted for drugs with ≥20 reports and a significant signal. Multivariable logistic regression analysis was employed to identify risk factors influencing reporting probability. Time-to-onset (TTO) was calculated from treatment initiation to event onset and analysed descriptively with median and Weibull shape parameters (WSP), and presented by Kaplan–Meier cumulative-incidence curves.

**Results:**

We identified 9,390 TD reports involving 399 drugs. Antipsychotics dominated the top 3 most frequently reported drugs: aripiprazole (n=2099), quetiapine (n=1121), and risperidone (n=910). Disproportionality analysis identified statistically significant TD signals for 57 drugs: antipsychotics (n=27, 47.4%), antidepressants (n=13, 22.8%), psychostimulants (n=4, 7.0%), and others (n=13, 22.8%). Sensitivity analyses confirmed significant positive aRORs for all evaluated agents. The strongest signals were observed for brexpiprazole (aROR=899.7) and cariprazine (aROR=732.6) among antipsychotics, fluoxetine (aROR=43.3) among antidepressants, and benztropine (aROR=467.3) among other classes. Age significantly influenced reporting risk; the effect of sex varied by drug, with higher risk in females for duloxetine and benztropine, and in males for citalopram and fluoxetine. TTO was shortest in patients aged<19 years (median=155.5 d). Second-generation antipsychotics (median = 421.0 d) and first-generation antipsychotics (626.0 d) exhibited longer TTOs than antidepressants (median=246.0 d). Weibull analysis indicated early-failure patterns for risperidone, olanzapine, lurasidone, haloperidol, and fluoxetine, while all other analyzed agents followed a random failure pattern over time.

**Conclusions:**

Our findings underscore significant TD risk associated with a broad pharmacologic spectrum, particularly with antipsychotics and antidepressants, and reveal distinct TTO profiles by drug class. These data support the need for individualized, long-term monitoring of TD risk during neuropsychiatric treatment.

**Disclosure of Interest:**

None Declared

